# Genome Sequences of Three Species D Adenoviruses Isolated from AIDS Patients

**DOI:** 10.1128/genomeA.01267-13

**Published:** 2014-02-06

**Authors:** Moustafa Alissa Alkhalaf, Yasir Mohammed A. Al Qurashi, Malcolm Guiver, Robert J. Cooper

**Affiliations:** a Virology Unit, Institute of Inflammation and Repair, the University of Manchester, Manchester, United Kingdom; b Public Health England, Manchester, United Kingdom

## Abstract

HAdVD23 and two novel adenoviruses, HAdVD60a and HAdVD62, isolated from feces of AIDS patients in Manchester, United Kingdom, have been sequenced. The HAdVD60a genome lacks the penton arginine-glycine-aspartic acid (RGD) motif and differs from HAdVD60 by a recombinant E3 region. HAdVD62 has penton, hexon, and fiber regions not previously found in other adenoviruses.

## GENOME ANNOUNCEMENT

More than 60 types of human adenoviruses have been identified and classified into seven species, A to G. They cause a variety of clinical features in the respiratory tract, eye, gastrointestinal tract, and other organs (1). These infections are usually mild in immunocompetent patients but can be severe in immunocompromised patients (2).

Three adenoviruses isolated from feces of AIDS patients in Manchester, United Kingdom (3, 4) included HAdVD23 and two novel viruses approved as new types by the Human Adenovirus Working Group, HAdVD60a (adenovirus D human/ENG/AIDS59/1992/60a [P37H20F60]) and HAdVD62 (adenovirus D human/ENG/AIDS25/1993/62 [P62H62F62]). HAdVD62 was from a patient with chronic diarrhea (but also infected with rotavirus), HAdVD23 from a patient without symptoms, and HAdVD60a from a patient whose symptoms are unknown to us.

Sequencing was carried out using the 454 GS FLX Titanium system (Roche) at the University of Liverpool Centre for Genomic Research. Briefly, the DNA was sheared and separated into single-stranded fragments. Adaptors were added to both ends of each fragment. Fragments were captured by DNA capture beads then amplified using emulsion PCR. Amplified fragments were sequenced by pyrosequencing. GS De Novo Assembler 2.0.00.20 (Roche) was used for genomic fragment assembly. The assembled contigs were ordered according to the completely sequenced HAdV-D genomes available in the GenBank.

The genome lengths of HAdVD23, HAdVD60a, and HAdVD62 were found to be 35,029 bp, 35,103 bp, and 35,127 bp, respectively. The overall base compositions were, for HAdVD23, 22.65% (A), 20.54% (T), 28.48% (G), and 28.33% (C); for HAdVD60a, 22.61% (A), 20.42% (T), 28.56% (G), and 28.41% (C); and for HAdVD62, 22.36% (A), 20.25% (T), 28.78% (G), and 28.61% (C). These data are similar to those of other members of HAdV-D.

Four early, two intermediate, and five late transcription units have been identified in the full-genome sequences of HAdVD23, HAdVD60a, and HAdVD62, which are similar to the organizations of other HAdV genomes, and each has 39 predicted coding sequences. The 5′ and 3′ ends of the HAdVD23, HAdVD60a, and HAdVD62 genomes have inverted terminal repeat (ITR) sequences of 148 bp, 147 bp, and 151 bp, respectively. The ITRs contain the transcription factor DNA binding site for nuclear factor III (NFIII) but not for NFI, which is absent from most species D adenoviruses. There are also binding sites for cellular factors such as stimulatory protein 1 (Sp1) and activating transcription factor (ATF).

The divergence between HAdV60a penton and HAdV-D37 was the result of a 78-nucleotide deletion from the HAdVD60a penton sequence, resulting in a 26-amino acid deletion including the RGD motif (Arg–Gly–Asp). This also occurs in HAdVD60 (5), and these are the only viruses within species D to have the RGD loop naturally deleted. The two viruses differ by a recombined E3 transcription unit. HAdVD62 has new penton, hexon, and fiber regions not previously found in other adenoviruses. HAdVD23 has 98% homology with the strain sequenced by Robinson et al. (6).

### Nucleotide sequence accession numbers.

The GenBank accession number for HAdVD23 is KF279629, for HAdVD60a is JN162672, and for HAdVD62 is JN162671.
